# Effects of Red Onion Peel Extracts on Oxidative Stress in *Caenorhabditis elegans*

**DOI:** 10.3390/biom16071024

**Published:** 2026-07-13

**Authors:** Héctor Palacios, Miguel Ángel Rodríguez, Nerea Abasolo, Adrià Ceretó, Sara Martinez de Cripan, Camille Malterre, Kevin Leonard, Helena Torrell, Antoni del Pino, Núria Canela, Job Tchoumtchoua, Marc Riu

**Affiliations:** 1 Eurecat, Centre Tecnològic de Catalunya, Center for Omic Sciences (COS), Joint Unit Universitat Rovira i Virgili-EURECAT, Unique Scientific and Technical Infrastructures (ICTS), 43204 Reus, Spain; hector.palacios@eurecat.org (H.P.); miguelangel.rodriguez@eurecat.org (M.Á.R.); nerea.abasolo@eurecat.org (N.A.); adria.cereto@ce.eurecat.org (A.C.); sara.martinez@eurecat.org (S.M.d.C.); helena.torrell@eurecat.org (H.T.); antoni.delpino@eurecat.org (A.d.P.); nuria.canela@eurecat.org (N.C.); 2 Biomass Valorization Platform, Extraction Department, CELABOR Srl, 4650 Herve, Belgium; camille.malterre@celabor.be (C.M.); kevin.leonard@celabor.be (K.L.)

**Keywords:** polyphenols, red onion peels, *Caenorhabditis elegans*, oxidative stress, subcritical water extraction, transcriptomics, metabolomics, lipidomics, multi-omics

## Abstract

Polyphenols are natural compounds with antioxidant properties that help prevent chronic diseases. Red onion (*Allium cepa*) peels are an underutilized source of polyphenols, offering a sustainable opportunity for the valorization of agricultural by-products. Polyphenol-rich extracts were obtained from red onion peels using subcritical water extraction and adsorption resin chromatography. Their biological effects were evaluated in *Caenorhabditis elegans* under oxidative stress conditions. A multi-omics approach integrating transcriptomics, metabolomics, and lipidomics was applied to assess molecular responses. The extract exhibited a high total phenolic content (>400 mg GAE/g) and strong antioxidant capacity, supporting a highly enriched polyphenolic profile. Survival assays confirmed the absence of toxicity at 100 µg/mL GAE. Transcriptomic analysis revealed 158 differentially expressed genes associated with stress response and metabolic regulation. Metabolomic profiling indicated a systematic reduction in amino acid levels alongside an increase in betaine in treated worms, suggesting adaptive metabolic reprogramming. Lipidomic analysis revealed significant lipid remodeling, characterized by decreased triglycerides, increased diacylglycerols, and changes in membrane phospholipids, indicating alterations in membrane composition and cellular responses associated with oxidative stress adaptation. Together, these results support a model in which red onion peel extracts induce coordinated transcriptional and metabolic adaptations associated with cellular resilience and stress adaptation. This study highlights the potential of agro-industrial by-products as functional bioactive ingredients and demonstrates the value of multi-omics approaches for uncovering system-level responses.

## 1. Introduction

Polyphenols are a diverse group of natural compounds widely recognized for their antioxidant properties, which contribute to the prevention of various diseases, including cardiovascular and neurodegenerative diseases, diabetes, and cancer. These compounds are abundant in a variety of plant-based foods [1], with red onions (*Allium cepa*) representing a particularly rich source [2]. Red onion peels, typically discarded as agricultural waste, contain higher concentrations of polyphenols than edible tissues, positioning them as a promising resource for developing high-value functional ingredients. Harnessing these polyphenols from onion peels not only provides a sustainable source of high-value compounds but also aligns with circular economy principles by reducing food waste.

The extraction and purification of polyphenols from onion peels can be effectively achieved using pressurized liquid extraction, such as subcritical Water Extraction (SWE) [3]. SWE offers several advantages over traditional extraction methods, particularly by using water under controlled temperature and pressure conditions, which eliminates the need for organic solvents and thus significantly reduces environmental impact. Additionally, by adjusting temperature and pressure, water’s polarity can be fine-tuned to optimize solubility and selectivity for targeted bioactive compounds, leading to high yields and improved extract purity while consuming less energy [4,5]. Following SWE, further concentration of polyphenolic compounds can be performed using chromatographic methods such as adsorption resin techniques (ART).

Despite increasing interest in plant-derived polyphenols, their systemic effects and underlying molecular mechanisms remain incompletely understood. To assess the bioactivity and potential therapeutic effects of these extracts, it is essential to conduct in vivo studies. In this context, *Caenorhabditis elegans* (*C. elegans*) offers an excellent model organism for such studies due to its well-characterized biology, genetic tractability, and established relevance in studies of aging, oxidative stress, and various diseases [6]. Moreover, this nematode has been widely used in toxicological screening and the evaluation of plant-based compounds, offering valuable insights into the mechanisms underlying their effects.

The aim of this study was to investigate the biological effects and underlying molecular mechanisms of polyphenol-rich extracts obtained from red onion peels in the model organism *Caenorhabditis elegans* under oxidative stress conditions. We hypothesized that these extracts would promote adaptive molecular responses associated with oxidative stress resistance through coordinated changes in gene expression, metabolite profiles, and lipid composition. To test this hypothesis, we applied an integrated multi-omics approach combining transcriptomics, metabolomics, and lipidomics. This integrative strategy enabled the identification of coordinated biological responses across multiple molecular layers, providing system-level insights into the mode of action of these bioactive compounds.

## 2. Materials and Methods

### 2.1. Plant Material

Red onion peels, *Allium cepa,* were provided by PROCECAM, a group of onion producers located in Castilla-La Mancha, Villarobledo, Albadete, Spain, and were shipped to Celabor by Cartif Technology Center (Boecillo, Valladolid, Spain). The peels collected in August 2021 were left in the field for 8 days to dehydrate before shipping.

### 2.2. Extraction of Polyphenols Using SWE

Red onion peels were subjected to SWE in a semi-pilot unit capable of performing pressurized liquid extraction by percolation. This equipment was specially designed for Celabor. Dried red onion peels were ground (using a 4 mm sieve) and introduced into a 6 L stainless steel insert. The insert was placed inside the reactor, and the system was sealed. Water was pumped and heated to 150 °C using a heat exchanger until the system was filled and reached the target pressure of 15 bar. Automatic valves were then closed, and the recirculation pump was activated to circulate the water through the extraction loop at a flow rate of 1000 g/min. Recirculation was maintained for 30 min, after which the liquid extract was cooled before the system was depressurized. The total liquid extract was then flushed into the collector using a nitrogen flow to drain the system, and the final extract was recovered from the extract collector.

### 2.3. Polyphenol Enrichment Using ART

The liquid red onion peel extract underwent an additional enrichment process using a hydrophobic adsorption resin to enhance its purity in batch mode. Following six optimization trials, including a resin screening step, Amberlite XAD4 was selected. Specifically, the liquid extracts were poured onto activated resins in a 25 L reactor at a 1:10 resin-to-liquid ratio and mechanically stirred for 16 h to maximize adsorption. The polyphenol-loaded resin was then recovered through filtration using a 200-mesh filter cloth and washed with fresh water for 1 h. The polyphenols were subsequently desorbed from the resin using 96% ethanol under mechanical stirring for 2 h, followed by filtration, vacuum evaporation, and spray-drying to obtain the enriched polyphenol extracts.

### 2.4. Characterization of the Polyphenol Extract

#### 2.4.1. Total Phenolic Content (TPC) by the Folin–Ciocalteu Method

The total phenolic content (TPC) was measured using the Folin–Ciocalteu method [7], with gallic acid serving as the reference compound. A 25 mg sample of the extract was dissolved in 5 mL of a 50:50 ethanol/water mixture and sonicated for 15 min to enhance solubility. The resulting solutions were diluted as required. To 100 µL of the diluted solution, 1.5 mL of distilled water, 400 µL of Folin–Ciocalteu reagent, 600 µL of 200 g/L Na_2_CO_3_, and 2.4 mL of distilled water were added. Each preparation was homogenized for 30 s using a vortex mixer operating at 2000 rpm (ZX3 Vortex Mixer, VELP Scientifica, Usmate Velate, Italy) and then incubated in the dark at room temperature (20–25 °C) for 2 h. Absorbance was then measured at 760 nm using a GENESYS 150 UV-Visible spectrophotometer (Thermo Fisher Scientific, Waltham, MA, USA). Quantification was performed using a gallic acid calibration curve ranging from 25 to 400 mg/L. The results were expressed as milligrams of gallic acid equivalents per gram of sample (mg GAE/g).

#### 2.4.2. Determination of Antioxidant Activity by the DPPH (2,2-Diphenyl-1-picrylhydrazyl) Method

The DPPH assay measures antioxidant activity based on electron transfer. DPPH is a stable free radical with a strong purple color that fades to pale yellow in the presence of electron-donating compounds. The extract was dissolved in methanol, and 5 mg of DPPH was dissolved in 25 mL of methanol. A calibration curve was prepared using gallic acid solutions ranging from 1 to 10 mg/L. In a microplate, 150 µL of each calibration standard and 150 µL of eight sample dilutions were dispensed into the wells. After the addition of 75 µL of the DPPH solution, the plate was incubated in the dark at room temperature (20–25 °C) for 30 min. Absorbance was then measured at 515 nm using an Infinite 200 series microplate reader (Tecan Group Ltd., Männedorf, Switzerland). Antioxidant activity was determined by comparing the IC_50_ values of the samples with those of gallic acid according to the following equation:

*Antioxidant power* (mg GAE/g) = *IC_50_ (gallic acid)/IC_50_ (sample)*

Results are expressed as milligrams of gallic acid equivalents per gram of dry extract (mg GAE/g).

#### 2.4.3. Quantitation of Major Polyphenols by UPLC-MS/MS

Polyphenols in the dry extract were identified and quantified using Ultra-High Performance Liquid Chromatography coupled with mass spectrometry on an Acquity UPLC Xevo-TQ system (Waters Corporation, Milford, MA, USA), equipped with an Acquity BEH Shield RP18 column. The gradient profile of the mobile phase consisted of an ammonium formate buffer (solvent A) and acetonitrile (solvent B): 0–7.20 min, 95–80% A; 7.20–10 min, 80–75% A; 10–11.5 min, 75–50% A; 11.5–12 min, 50–0% A; 12–12.5 min, 0% A (isocratic); 12.5–14 min, 0–95% A; 14–16.5 min, 95% A (isocratic), with a constant flow rate of 0.5 mL/min. The column temperature was set at 40 °C, and sample vials were maintained at 15 °C during analysis. An injection volume of 3.5 µL was used, and the analyses were performed using MassLynx V4.2 software (Waters). Polyphenols were identified based on their *m*/*z* values and fragmentation patterns and compared with an internal database containing more than 100 standards. Quantification was performed using calibration curves of external standards with concentrations ranging from 0.25 to 10 mg/L. The results were expressed in mg/g.

### 2.5. Culturing of Nematodes

*C. elegans* is small and easy to cultivate, making it suitable for laboratory studies using *Escherichia coli* (*E. coli*) as a food source. Wild-type *C. elegans* strain N2 and *Escherichia coli* OP50 were obtained from the Caenorhabditis Genetics Center (CGC). A single isolated colony of *Escherichia coli* OP50 was selected from a Luria–Bertani (LB) agar plate and inoculated into sterile LB liquid medium. The culture was incubated overnight at 37 °C with continuous shaking at 180 rpm to ensure adequate aeration and bacterial growth. The resulting overnight culture was used for all subsequent experimental procedures.

This nematode can be grown at different temperatures, but 20 °C provides the highest fertility rates. Therefore, worms were cultured at 20 °C in nematode growth medium (NGM) with *E. coli* OP50 as a food source.

To ensure that all worms were at the same developmental stage, worms were synchronized. Worms and eggs were collected from plates using M9 buffer and transferred into 15 mL Falcon tubes. The samples were centrifuged at 1500 rpm for 1 min at 20 °C. The supernatant was carefully removed, leaving 1 mL of buffer. To remove *E. coli*, this step was repeated twice more. After the final wash, worms and eggs were kept in 1 mL of solution and prepared to be synchronized.

For synchronization, a mixture of 1 mL of bleach solution, 2.5 mL of 1 M NaOH, and 0.5 mL of H_2_O was added to a 15 mL Falcon tube and vigorously mixed for 3 min. After this period, the previously mentioned washing step was repeated three more times to dilute the synchronization buffer. The sample was then left rotating overnight at 15 °C to allow egg hatching. This process resulted in a synchronized L1 worm population.

To develop the oxidative model, L1-stage worms were placed on NGM plates and incubated for 4 days at 15 °C to reach the young adult stage. Worms were fed with heat-inactivated *E. coli* OP50, and treatments were applied at this stage. Heat-inactivated bacteria were used to minimize potential metabolic contributions from actively growing bacteria and to ensure that the observed molecular changes were primarily attributable to the treatment and host response. On the 4th day, the plates were placed in an incubator at 35 °C for 2 h to induce oxidative stress. To further support the suitability of this model, previous studies have reported ROS accumulation in *C. elegans* exposed to 35 °C. Furthermore, intracellular ROS levels were previously evaluated under the same experimental conditions used in this study, revealing a progressive increase during heat exposure and confirming the induction of oxidative stress. The complete results are provided in Table S1 (Supplementary Materials). Afterward, the samples were collected in Eppendorf tubes and stored at −80 °C for further analysis [8].

Oxidative stress, toxicity, and control treatments (under the same conditions as oxidative stress, except without extract) were carried out in triplicate. Depending on the downstream analysis, approximately 40 worms per biological replicate were used for the toxicity assay, 1000 worms per replicate for transcriptomic analyses, 5000 worms per replicate for lipidomic analyses, and 10,000 worms per replicate for metabolomic analyses. All experimental groups were fed under identical conditions using heat-inactivated *E. coli* OP50.

### 2.6. Transcriptomics by Next Generation Sequencing (NGS)

High-throughput sequencing techniques, such as RNA sequencing (RNA-Seq), have become powerful tools for transcriptome studies, offering a comprehensive and unbiased analysis of gene expression profiles. Unlike microarrays, which are limited to detecting known transcripts, next-generation sequencing (NGS) technologies provide an in-depth view of the transcriptome from any given tissue or cell type.

In this study, total RNA was extracted, and its integrity was assessed using the TapeStation System (Agilent Technologies, Santa Clara, CA, USA).

Sequencing libraries were prepared using the Illumina Stranded mRNA Prep Kit (Cat. No. 20040534), following the manufacturer’s protocol. Paired-end sequencing (2 × 76 bp) was performed on the Illumina NextSeq 2000 platform. Raw reads were aligned to the *C. elegans* reference genome (GCF_000146045.2) using HISAT2 (v2.2.1), and transcript assembly was conducted with StringTie (v2.2.1). Differential expression analysis was performed using DESeq2 v1.34.0.

### 2.7. Metabolomic Analysis by ^1^H NMR Spectroscopy

Samples were initially lyophilized using a FreeZone 2.5 L freeze dryer (Labconco, Kansas City, MO, USA) equipped with a −84 °C condenser and operated under vacuum according to the manufacturer’s recommended conditions for at least 15 h. Once dried, a methanol:water (8:1, *v*/*v*) mixture was added to extract the polar phase. The samples were then vortexed and sonicated on ice for 10–15 min until complete homogenization. To separate the polar phase, the samples were centrifuged at 15,000 rpm (20,000× *g*) for 10 min at 4 °C. The supernatant was transferred to a new Eppendorf tube and dried using a SpeedVac SPD2030 vacuum concentrator (Thermo Fisher Scientific, Waltham, MA, USA), while the pellet was discarded.

For NMR measurements, 600 µL of phosphate-buffered saline (PBS) in D_2_O (pH 7.4, 0.05 M) containing 1.48 mM trimethylsilylpropanoic acid (TSP, final concentration 1 mM) was added to the dried extract. The solution was vortexed and transferred into a 5 mm outer diameter (o.d.) NMR tube. The resulting mixture was inspected to ensure a clear dispersion; if necessary, an additional centrifugation step (14,000 rpm, ~14,000× *g*, 5 min, room temperature (20–25 °C)) was performed before transferring the sample to the NMR tube (Eretic Signal 1.3214 mM).

^1^H NMR spectra were recorded at 300 K using an Avance III 600 spectrometer (Bruker BioSpin GmbH, Rheinstetten, Germany) operating at a proton frequency of 600.20 MHz with a 5 mm PABBO broadband gradient probe. Aqueous samples were analyzed under standard conditions (procno 11). One-dimensional (1D) ^1^H-NMR spectra were acquired using the nuclear Overhauser effect spectroscopy (NOESY) presaturation sequence (RD–90–t_1_–90–t_m_–90–ACQ) to suppress the residual water signal. The mixing time was set to 100 ms, and solvent presaturation was applied with an irradiation power of 125 mW during the recycling delay (RD = 5 s) and mixing time. The 90° pulse length was calibrated individually for each sample, ranging from 9.60 to 10.38 µs. The spectral width was set to 9.6 kHz (16 ppm), and 256 transients were collected into 64 K data points for each ^1^H spectrum.

An exponential line broadening of 0.3 Hz was applied before Fourier transformation. The resulting frequency-domain spectra were manually phased, baseline-corrected, and referenced to TSP (*δ* = 0 ppm) using TopSpin software (v3.6, Bruker). Metabolite identification was performed by comparing acquired ^1^H NMR spectra with reference spectra from the AMIX metabolic profiling database (Bruker), the Human Metabolome Database (HMDB), and the Chenomx Suix 8.5 Professional database. Additional metabolite assignments were confirmed using ^1^H–^1^H homonuclear correlation spectroscopy (COSY), total correlation spectroscopy (TOCSY), and ^1^H–^13^C heteronuclear single quantum coherence (HSQC) two-dimensional (2D) NMR experiments, as well as by comparison with pure compound standards analyzed in-house.

Following pre-processing, specific ^1^H NMR spectral regions corresponding to identified metabolites were integrated using the AMIX software package (v3.9). Curated spectral regions were exported to an Excel spreadsheet to assess the robustness of ^1^H NMR signals and to estimate relative metabolite concentrations. Total protein content was determined by the Bradford assay.

### 2.8. Lipidomic Analysis by LC-MS/MS

Lipidomic profiling was conducted using an Agilent 6546 UHPLC-qTOF system (Agilent Technologies, Santa Clara, CA, USA) equipped with a Kinetex EVO C18 column (2.6 µm, 2.1 mm × 100 mm; Phenomenex, Torrance, CA, USA). Approximately 5000 *C. elegans* worms per sample were lyophilized under the same conditions described in Section 2.7 and reconstituted in 100 µL of phosphate-buffered saline (PBS). Lipids were extracted using a modified Folch method (chloroform:methanol, 2:1 *v*/*v*), followed by the addition of 0.8% NaCl and sonication. After centrifugation (15,000 rpm, 10 min, 4 °C), the organic phase was collected, evaporated under nitrogen, and reconstituted in methanol:methyl tert-butyl ether (9:1 *v*/*v*). Internal standards (SPLASH Lipidomix, Avanti Polar Lipids, Alabaster, AL, USA) were added prior to extraction for normalization. Total protein concentration was determined by the Bradford assay.

Chromatographic separation was achieved using a quaternary solvent system (water, methanol, 2-propanol, and water with 200 mM ammonium formate and 2% formic acid). A gradient elution ranging from 50% aqueous to 100% organic over 11.5 min was applied to separate lipid classes, optimized for sequential elution of lysophospholipids, sphingomyelins, phospholipids, diacylglycerols, triglycerides, and cholesteryl esters.

The flow rate was 0.6 mL/min, the column temperature was 40 °C, and the injection volume was 0.2 µL. The qTOF mass spectrometer operated in both positive and negative electrospray ionization (ESI) modes, scanning *m*/*z* 300–1000. Instrument parameters included gas temperature 225 °C, gas flow 11 L/min, nebulizer pressure 35 psi, sheath gas temperature 300 °C, sheath gas flow 12 L/min, capillary voltage 3500 V, and nozzle voltage 500 V.

Data acquisition and lipid annotation were performed using Agilent MassHunter software (version 11.0) and Lipid Annotator (version 1.0). Lipid identification was based on exact mass, retention time, and MS/MS fragmentation patterns, with reference to LIPID MAPS and HMDBs. An iterative MS/MS method was applied for structural confirmation. Lipid species were quantified and classified into major classes, including phosphatidylcholines (PC), phosphatidylethanolamines (PE), sphingomyelins (SM), diacylglycerols (DG), and triglycerides (TG). For some lipid compositions, more than one chromatographic feature with identical MS/MS spectra was detected. Given the inability to unambiguously assign structural differences, these features were reported separately without assuming distinct molecular species.

### 2.9. Statistical Analysis

#### 2.9.1. Differential Gene Expression Analysis

Differential gene expression was assessed using the DESeq2 v1.34.0 package [9]. This method models RNA-Seq count data using a negative binomial distribution and estimates biological variability through shrinkage of dispersion estimates. Expression changes are reported as log2 fold changes, and statistical significance is determined using the Wald test. *p*-values were adjusted for multiple testing using the Benjamini–Hochberg procedure to control the false discovery rate (FDR).

#### 2.9.2. Multivariate and Multi-Omics Data Integration Analysis

Multivariate data analysis was conducted using the mixOmics v6.8.5 R package [10] to explore relationships both within and across omics layers. Three datasets were integrated: TPM-normalized transcriptomics (158 genes, statistically significant across conditions), auto-normalized metabolomics (30 metabolites), and lipidomics (296 lipids), with missing values in the lipidomics data imputed using the NIPALS algorithm.

Unsupervised principal component analysis (PCA) was first applied to each dataset to assess overall structure, variability, and potential outliers. Multi-omics integration was then conducted using sparse multiblock partial least squares (multiblock-sPLS), with lipidomics as the outcome matrix and transcriptomics and metabolomics as predictors. The design matrix specified weak (0.1) correlations between blocks. Variable selection for sparse models was guided by prior exploratory multiblock-PLS analyses, selecting 30 and 20 transcriptomic features, 10 and 9 metabolomic features, and 40 and 20 lipidomic features for components 1 and 2, respectively. The PLS and sPLS analyses were used to explore coordinated patterns of variation across omics layers and to identify molecular features associated with lipidomic variation.

#### 2.9.3. Additional Multivariate Analysis

To identify significant changes across the studied conditions, additional analyses were conducted using MetaboAnalyst 6.0 (https://www.metaboanalyst.ca/) (accessed on 23 June 2026). Metabolomic and lipidomic datasets were normalized, log10-transformed, and autoscaled (mean-centered and divided by the standard deviation of each variable). Statistical analyses included principal component analysis (PCA) and complementary approaches for group comparison, including heatmap-based visualization.

Generative AI assistance. During manuscript preparation, ChatGPT (OpenAI, GPT-5.5) was used exclusively to assist with language editing, grammar correction, improvement of text clarity and readability, and refinement of the scientific writing. All scientific content, data interpretation, conclusions, and final editorial decisions were reviewed, verified, and approved by the authors, who take full responsibility for the content of the manuscript.

## 3. Results

### 3.1. Characterization of the Enriched Red Onion Peel Extract

After SWE, the extraction yield (mass of the dry extract divided by the mass of the dry feedstock) of the red onion peels was 24.05%. However, after ART, only 26.41% of the extract was retained on the chromatographic column as an enriched extract. Hence, the final extraction yield of the enriched extract was 6.35%. Table 1 summarizes the total phenolic content and antioxidant power of the extract.

Table 2 reports the polyphenolic composition of the enriched extract, with quercetin and spiraeoside (quercetin glycosylated form) being the major components. Protocatechuic acid, quercetin-7-O-glucoside, isoquercitrin, isorhamnetin, and tamarixetin were also present in lower concentrations.

### 3.2. Toxicity Assessment

**Figure 1:**
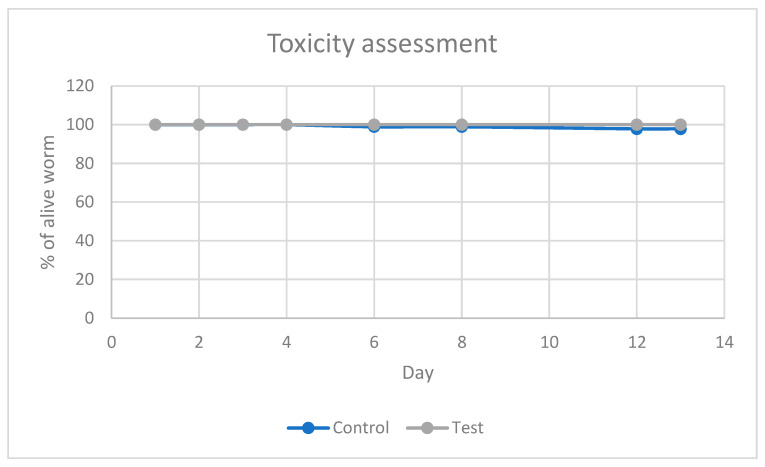
Toxicity assessment. The survival rate of worms treated with polyphenol was comparable to that of the control group without polyphenol (*n* = 3).

A preliminary toxicity assessment was performed to evaluate the safety of the treatment. A concentration of 100 µg/mL GAE was selected for the analysis. After 13 days, no significant differences were observed between the control and the treated groups (Figure 1), indicating the absence of treatment-related toxicity under the experimental conditions tested. This concentration was therefore used for subsequent experiments.

### 3.3. Transcriptomics

Differential expression analysis revealed a robust and coordinated transcriptional response to polyphenol treatment. Genes associated with stress responses, metabolic regulation, and cellular adaptation were prominently represented, suggesting activation of conserved protective pathways.

**Figure 2:**
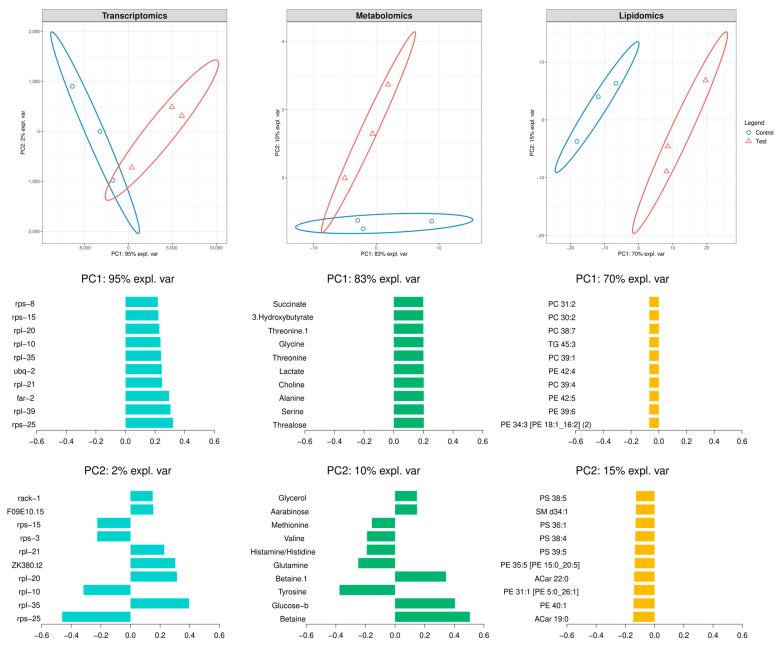
Principal Component Analysis (PCA) of transcriptomics, metabolomics, and lipidomics profiles from *C. elegans* samples comparing control and test groups. The score plots show a clear separation between groups, with PC1/PC2 explaining 95% and 2% (transcriptomics), 83% and 10% (metabolomics), and 70% and 15% (lipidomics) of the variance, respectively. Ellipses depict group dispersion (95% confidence). Bar plots display the top positive/negative loadings for PC1 and PC2 in each omics layer, highlighting the variables contributing most to group separation. Three independent biological replicates were analyzed for each experimental condition (*n* = 3).

PCA (Figure 2) was performed to assess the overall variance in gene expression profiles between the experimental groups. The resulting PCA plot revealed a clear separation between the control and polyphenol-treated groups, indicating distinct transcriptional profiles. The clustering of samples within each group, along with the separation along the principal components, suggests that the polyphenol treatment induces consistent and measurable changes in gene expression. The presence of confidence ellipses further supports the reproducibility and group-specific variance of the data.

**Figure 3:**
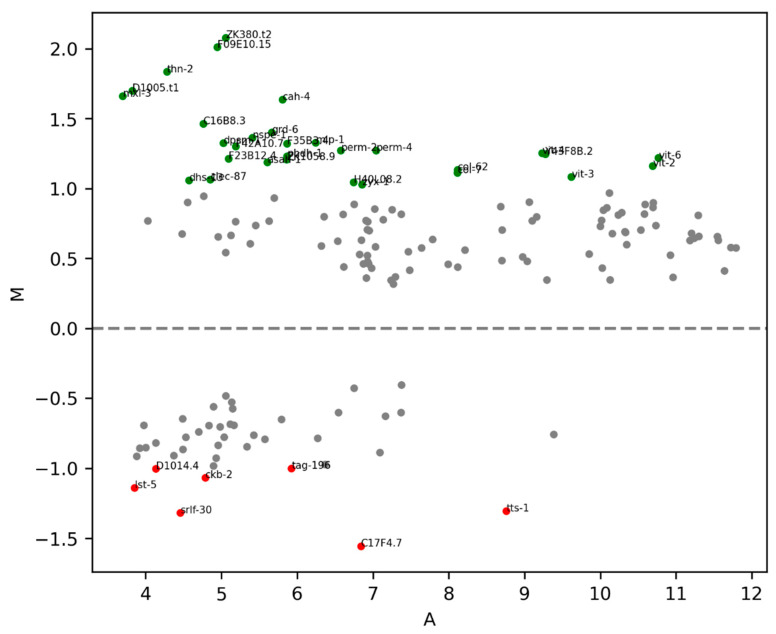
MA plot showing the distribution of gene expression changes between the test (polyphenol-rich extract-treated) and the control group. Each point represents a gene, with the *x*-axis indicating average expression (A) and the *y*-axis representing log_2_ fold change (M). Green and red points denote significantly upregulated and downregulated genes, respectively (*p*adj < 0.05), while grey points represent non-significant changes. Labeled genes highlight those with the most pronounced expression differences. The complete results are provided in Table S2 (Supplementary Materials). Differential expression analysis was performed using three independent biological replicates per experimental condition (*n* = 3).

Differential expression analysis using DESeq2 identified a set of significantly differentially expressed genes between treated and control samples. Genes with an adjusted *p*-value (*p*adj) below 0.05 were considered statistically significant, as shown in Figure 3. Among these, both upregulated and downregulated genes were identified, indicating a clear transcriptional response to the treatment. A total of 158 genes were found to be significantly differentially expressed (*p*adj < 0.05), of which 116 were upregulated and 42 were downregulated in the polyphenol-treated group compared to the control.

Rather than isolated gene-level changes, the observed transcriptional profile points to a system-wide reprogramming of cellular function, consistent with an adaptive response to oxidative stress conditions.

### 3.4. Metabolomic Analysis by ^1^H NMR Spectroscopy

Metabolomic profiling revealed distinct and coherent metabolic shifts between control and treated groups. The overall reduction in amino acid levels, together with the increase in betaine, suggests a redistribution of metabolic resources towards osmoprotection and cellular stability.

PCA (Figure 2) was performed to evaluate global metabolic variation between the experimental groups. The PCA plot showed a clear separation between the control group and the test group (polyphenol extract-treated), indicating distinct metabolomic signatures associated with the treatment. Samples clustered consistently within each group, and the separation along the principal components, particularly PC1, which accounted for the majority of explained variance, reflects coordinated metabolic changes induced by the extract. The confidence ellipses further illustrate the stability of the group-specific profiles and support the reproducibility of the metabolomic response.

These changes are consistent with a metabolic adaptation associated with the cellular response to stress, rather than a simple perturbation of metabolic homeostasis.

The complete results are provided in Table S3 (Supplementary Materials).

### 3.5. Lipidomics

Lipidomic analysis revealed extensive remodeling of lipid classes, with a reduction in triglycerides and changes in membrane phospholipids, including PC and PE. This pattern suggests remodeling of membrane components.

Such remodeling is consistent with improved membrane integrity and cellular adaptability under stress conditions, highlighting the central role of lipid metabolism in mediating the extract’s biological effects.

PCA (Figure 2) was performed using 296 lipid variables to evaluate global lipidomic variation between the experimental groups. The PCA plot showed a clear separation between the control group and the test group (polyphenol extract-treated), indicating distinct lipidomic signatures associated with the treatment. Samples clustered consistently within each group, and the separation along the principal components, particularly PC1, which accounted for most of the explained variance (70%), reflects coordinated shifts in lipid composition induced by the extract. The confidence ellipses further illustrate the stability of the group-specific profiles and support the reproducibility of the lipidomic response.

In this model, PC1 clearly separated the test samples from the control samples, highlighting treatment-associated changes in neutral lipids and membrane-related species.

**Figure 4:**
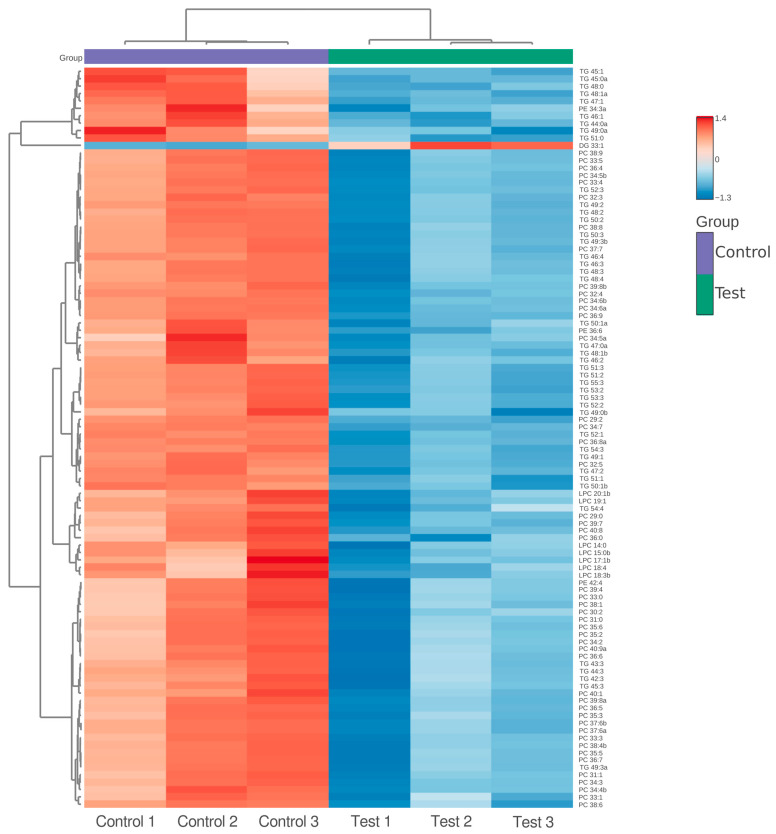
Heatmap showing significant lipidomic differences between the test (polyphenol extract-treated) and the control groups. The 100 most relevant lipid species are displayed. Lipid names are expressed as classes (sum of composition; constituent species), following shorthand lipid nomenclature. Color scale represents normalized abundance values (red = higher, blue = lower). Three independent biological replicates were included for each experimental condition (*n* = 3).

The heatmap generated (Figure 4), representing the 100 most relevant lipid species, identified significant lipid modifications, including diacylglycerols (DG), phosphatidylcholines (PC), phosphatidylethanolamines (PE), phosphatidylserine (PS), sphingomyelins (SM), and triacylglycerols (TG). The heatmap was generated after normalization to protein content, log10 transformation, and autoscaling.

The complete results are provided in Table S4 (Supplementary Materials).

### 3.6. Holistic Interpretation

**Figure 5:**
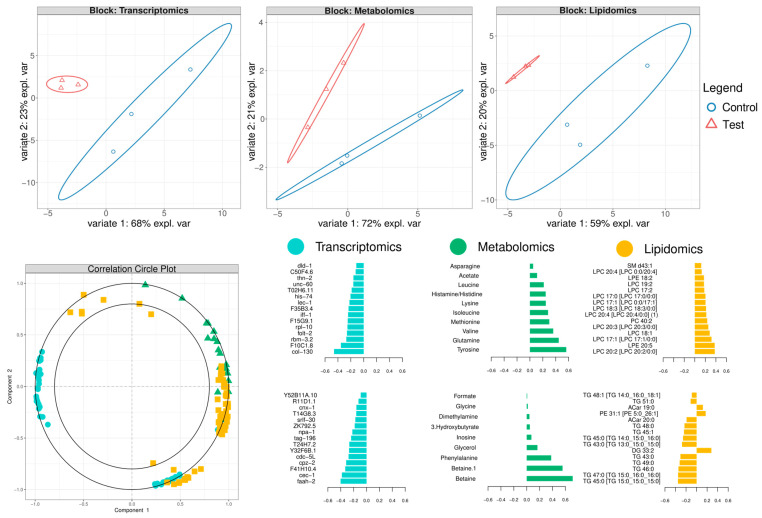
Multiblock sPLS integration of transcriptomics, metabolomics, and lipidomics. Score plots for each omics block (top row) show clear separation between control (blue circles) and test (red triangles) samples. Three independent biological replicates were analyzed for each experimental condition (*n* = 3).

In this study, we integrated transcriptomic, lipidomic, and metabolomic datasets to explore the shared structure across omics layers and identify coordinated molecular patterns. Using a multiblock sparse Partial Least Squares (sPLS) approach, we found that two components effectively summarized the variability within each omics block. Component 1 explained 68% of the variance in transcriptomics, 72% in metabolomics, and 59% in lipidomics, while Component 2 accounted for an additional 23%, 19%, and 20%, respectively. Together, the first two components captured most of the structure present in each dataset (see Figure 5). This dimensionality reduction revealed a consistent underlying multivariate organization. It enabled the identification of key transcript, metabolite, and lipid features that jointly contribute to the separation between control and test samples. The correlation circle plot (bottom left) illustrates cross-omics relationships among variables contributing to the multiblock model. Bar plots (bottom-right) display the most relevant features with the highest loadings on the first two components for each omics layer, highlighting the molecular variables driving group discrimination. The sPLS loadings revealed distinct patterns across transcriptomic, metabolomic, and lipidomic datasets. Although Component 1 emerged as the most integrative axis across the multi-omics dataset, capturing the largest proportion of explained variance, the metabolomics data showed a clearer group separation along Component 2. This indicates that Component 1 primarily captures the underlying lipidomic structure, whereas the discrimination between groups is more strongly reflected in Component 2.

The bar plots display a subset of variables selected by the sPLS model for each component. In contrast, the full loading table contains the complete set of loading values for all variables and components. Regarding the loadings, Component 1 emerged as the most integrative axis, with strong contributions from all three omics layers. Transcriptomic variables such as *rbm-3.2* [11], *folt-2* [12], *rpl-10*, and *nurf-1* [13] showed high negative loadings, while metabolites including valine, glutamine, and tyrosine were positively loaded. Lipidomic features such as LPC 20:2, LPE 20:5, and LPC 17:1 also contributed significantly.

Component 2 revealed more specific molecular responses. Among the transcriptomic variables, *faah-2* [14] and *cec-1* [15] exhibited the strongest negative loadings, whereas other genes, including *pfas-1* [16], contributed to a lesser extent. In the metabolomic dataset, betaine was the variable most strongly associated with this component, followed by phenylalanine, while glycerol showed a more moderate positive contribution. Regarding the lipidomic profile, variables such as TG 45:0, TG 47:0, TG 46:0, TG 49:0, and TG 43:0 (predominantly triacylglycerols) were negatively associated with this component. In contrast, DG 33:2 showed a positive association, whereas other diacylglycerol species (DG 40:10, DG 37:2, and DG 36:3) exhibited only minor positive contributions.

The loading bar plots highlight the variables that contribute most strongly to Component 1 within each omics block, revealing the molecular features that drive the main axis of variation in the multiblock sPLS model.

In the transcriptomic block, Component 1 was dominated by a subset of negatively loaded genes, particularly *col-130*, *F10C1.8*, *rbm-3.2*, *folt-2*, *rpl-10*, *F15G9.1*, and *iff-1*. The absence of strongly positive transcriptomic contributors suggests that these negatively associated genes largely drove the transcriptomic variation captured by this component.

In the metabolomic block, Component 1 was characterized by strong positive contributions from several amino acids, particularly tyrosine, glutamine, valine, methionine, isoleucine, lysine, histamine/histidine, and leucine. The coordinated contribution of these metabolites indicates that amino acid metabolism represents a major source of variation captured by this component.

In the lipidomic block, Component 1 was mainly driven by positive loadings of lysophospholipid species, particularly LPC 20:2, LPE 20:5, LPC 17:1, LPC 18:1, and LPC 20:3. Additional contributions were observed for phosphatidylcholine species such as PC 40:2. Overall, the lipidomic variation captured by this component was predominantly associated with lysophospholipids and phosphatidylcholines.

## 4. Discussion

This study provides a system-level characterization of the biological effects of red onion peel-derived polyphenols in *C. elegans* using an integrative multi-omics approach. Rather than isolated molecular changes, our results reveal a coordinated adaptive response involving transcriptional regulation, metabolic reprogramming, and lipid remodeling.

### 4.1. Characterization of the Enriched Red Onion Peel Extract

As shown in Table 1, the enriched extract exhibited a high total polyphenol content, exceeding 400 mg GAE/g. The DPPH assay also showed a high antioxidant capacity, close to 200 mg GAE/g, comparable to values reported for well-known plant extracts, such as tea extracts.

### 4.2. Toxicity Assessment

The absence of significant differences in survival between the treated and control groups suggests that the selected concentration (100 µg/mL GAE) does not exert toxic effects on *C. elegans*. This finding supports its suitability for further assays and indicates that the extract can be tested at this dose without compromising organism viability.

### 4.3. Transcriptomics

At the transcriptomic level, the clear separation observed in the PCA and the large number of differentially expressed genes suggest that polyphenol treatment induces consistent transcriptional changes. The modulation of genes associated with stress responses and metabolic regulation suggests the activation of conserved defense mechanisms. These changes indicate that the extract does not merely exert antioxidant activity but instead triggers endogenous cellular adaptation pathways.

More specifically, several genes stand out for their potential biological relevance in the context of oxidative stress and cellular adaptation. The upregulated gene *thn-2* is associated with oxidative stress response mechanisms, suggesting a protective role of polyphenol treatment [17,18]. Similarly, *grd-6* [19] and *mxl-3* [20] may be involved in transcriptional regulation and signaling pathways that mediate stress adaptation. The gene *nspe-1* also showed strong induction and likely encodes a nematode-specific neuropeptide involved in metabolic regulation and density-dependent intracellular signaling [21]. On the other hand, several downregulated genes, such as *dpy-18*, *tts-1* and *ckb-2* are linked to structural integrity and energy metabolism, respectively, indicating a possible shift in physiological priorities under polyphenol exposure. For instance, *ckb-2* encodes a choline kinase, an enzyme that has been associated with tumor development and metastasis in *C. elegans* [22]. The gene *tts-1* has been linked to longevity and stress response pathways. Therefore, its downregulation could indicate an altered stress response, possibly due to the protective effects of polyphenols [23]. *dpy-18* encodes the α-subunit of prolyl-4-hydroxylase in *C. elegans*, an enzyme required for collagen hydroxylation, which is essential for maintaining cuticle structure. The observed downregulation of *dpy-18* may reflect a decreased demand for structural maintenance, potentially due to improved cellular stability or reduced tissue turnover under polyphenol treatment [24]. These findings support the hypothesis that polyphenol treatment modulates gene expression patterns associated with stress resilience and metabolic regulation in *C. elegans*.

These transcriptional changes may reflect an enhanced capacity to maintain cellular homeostasis under stress conditions and support adaptive responses associated with stress resilience and metabolic regulation [25].

### 4.4. Metabolomic Analysis by ^1^H NMR Spectroscopy

Consistent with the transcriptomic interpretation, metabolomic data revealed a redistribution of metabolic resources, particularly reflected in overall decreased amino acid levels and increased betaine levels. Betaine is a well-known osmoprotectant and methyl donor, and its accumulation suggests an enhanced capacity for cellular protection and metabolic flexibility under stress conditions. This pattern is indicative of active metabolic reprogramming rather than passive metabolic disruption.

As shown in Figure 2, metabolomic PCA revealed that the samples treated with the polyphenol-rich extract exhibited a distinct metabolic shift in *C. elegans*, primarily captured by PC1, which explained 83% of the variance. Test samples clustered together and were characterized by high loadings of succinate, 3-hydroxybutyrate, lactate, choline, and several amino acids, suggesting enhanced activity of central carbon metabolism and mitochondrial-associated pathways [26]. The prominent contribution of organic acids and amino acids to the metabolic separation indicates adjustments in energetic and biosynthetic fluxes.

PC2 (10% of the variance) further differentiated the groups and highlighted a set of metabolites strongly associated with the treatment, most notably betaine, along with glucose-derived intermediates, branched-chain amino acids, aromatic amino acids, and glycerol [27]. Betaine, in particular, is a well-known osmoprotectant and methyl donor, and its increased abundance may reflect enhanced cellular protection mechanisms, methylation capacity, or compatible-solute dynamics in response to the treatment [28]. In contrast, glutamine exhibited a negative loading and was more closely associated with the control samples, potentially indicating differences in nitrogen handling or anaplerotic flux between groups.

Together, these data reveal a coordinated metabolic remodeling triggered by the polyphenol-rich extract, involving central energy metabolism, amino acid turnover, osmoprotective mechanisms, and membrane-related pathways. This reorganization may support cellular adaptation to stress or increased metabolic demands in treated animals.

### 4.5. Lipidomics

The lipidomic heatmap shows a clear and consistent separation between control and test samples, with most lipids displaying lower abundance in the test group. This global pattern is reflected in the strong clustering of samples by condition. Lipid composition and its modifications provide insights into oxidative stress and metabolic processes [29,30]. Certain plant extracts have shown positive effects against oxidative stress [31], reducing lipid and TG levels [32,33,34].

The reduction in TG species observed aligns with polyphenol-associated antioxidant activity, while the increase in DG levels is consistent with aging-related lipid metabolism in *C. elegans* [35,36,37].

Phospholipids (PLs) are asymmetrically distributed in membranes. The most abundant membrane PLs are PC, PE, PS, PI, and cardiolipin [38]. Changes in sphingomyelin (SM) species were also observed in treated samples. Although sphingomyelin metabolism in *C. elegans* has been associated with aging and oxidative stress, sphingomyelins are also key components of cellular membranes. They play important roles in maintaining membrane stability and regulating signaling pathways. Previous studies have shown that modulation of sphingomyelin metabolism affects development, stress resistance, and lifespan in *C. elegans*. Moreover, changes in sphingomyelin composition have also been linked to membrane remodeling and cellular adaptation to stress. Therefore, the biological significance of the changes in sphingomyelin composition observed in the present study should be interpreted within the broader context of the coordinated transcriptomic, metabolomic, and lipidomic responses induced by the treatment [39,40].

Both PC and PE are essential phospholipids involved in maintaining mitochondrial membrane integrity and cellular homeostasis [41,42]. In *Caenorhabditis elegans*, age-related declines in PC and PE synthesis have been linked to mitochondrial dysfunction and increased oxidative stress. In our experimental model, changes in PC and PE suggest remodeling of membrane phospholipid composition, potentially related to oxidative stress adaptation. These findings align with evidence showing that restoring PC levels through dietary supplementation alleviates mitochondrial fragmentation and enhances metabolic plasticity.

Similarly, alterations in PE composition may contribute to mitochondrial dynamics and stress adaptation, likely through the hormetic activation of protective pathways. Together, these observations suggest that changes in membrane phospholipid composition may contribute to cellular adaptation to oxidative stress and to the preservation of mitochondrial integrity during aging [43,44].

Phosphatidylserine (PS), a key phospholipid component of cellular membranes, has been shown to play a significant role in modulating the oxidative stress response in *Caenorhabditis elegans*. Dietary supplementation with PS significantly increases resistance to oxidative stress and extends lifespan in *C. elegans*, albeit with a trade-off of reduced fertility [45]. This protective effect is mediated by a hormetic mechanism, in which low-level stress induced by PS activates endogenous defense pathways. Notably, the longevity-promoting effects of PS require the transcription factor DAF-16, a central regulator of the insulin/IGF-1 signaling pathway, which is known to govern stress resistance and aging [46].

Overall, lipidomic alterations further support the model described by gene expression and metabolic profiling. The observed decrease in triglycerides, combined with changes in membrane phospholipids, including PC and PE, indicates substantial lipid remodeling associated with membrane maintenance and functional adaptation. Phospholipids are critical for maintaining mitochondrial integrity and cellular homeostasis, and their remodeling is consistent with cellular responses associated with oxidative stress adaptation.

### 4.6. Holistic Interpretation

Importantly, these multi-layered changes converge towards a unified biological interpretation: the extract induces a transition from a storage-oriented metabolic state to a protective and adaptive state. This transition is a hallmark of adaptive stress-resistance mechanisms and has been widely associated with improved organismal resilience and, in some contexts, longevity in *C. elegans*.

While no differences in survival were observed under the conditions tested, the molecular multi-omics signatures observed are consistent with adaptive responses associated with oxidative stress. These findings suggest that the extract appears to promote stress adaptation and cellular resilience, consistent with a protective response to oxidative stress.

The holistic interpretation of the different omics data indicates that these molecular features could serve as potential biomarkers of the applied treatment, as they are all related to energy metabolism, cellular signaling, or stress response. Together, the findings suggest that the treatment induces coordinated molecular changes, particularly affecting pathways linked to oxidative stress resilience.

The correlation circle plot (Figure 5) shows the relationship between variables from each omics layer and the first two components. Variables positioned near the outer circle display strong correlations with the components and therefore exert a greater influence on the multivariate structure. In this representation, transcriptomic variables cluster predominantly on the left side of Component 1, indicating that most genes have a negative correlation with the main axis. Both metabolomics and lipidomics occupy mainly the right side of Component 1, reflecting their strong positive association with this component. The spatial distribution underscores complementary layer-specific contributions, with transcriptomic variables exhibiting negative alignment on Component 1 in contrast to the predominantly positive alignment of metabolomic and lipidomic variables.

Several variables identified in both Component 1 and Component 2 may serve as potential biomarkers of the treatment’s effects. Collectively, the metabolomic and lipidomic signatures support coordinated changes in osmoregulation, energy metabolism, and cellular adaptation to stress, consistent with the patterns described in the individual omics analyses.

In addition to the previously discussed features, several other transcriptomic markers showed notable changes in their contribution to the components, suggesting broader biological implications. The upregulated gene *col-130* [47], which is involved in structural integrity, contributed negatively to the components. A negative contribution was also observed for the upregulated gene *iff-1* [48], which is required for germ cell proliferation, gametogenesis, and localization of the P-granule component PGL-1. Furthermore, *lec-1* [49], a major galectin in *C. elegans* with a protective role against oxidative stress, and *thn-2* [50], associated with the immune response, were among the transcripts with relevant loadings.

Although these findings suggest potential roles in structural remodeling, reproductive processes, and stress defense, interpreting these transcriptomic patterns remains complex and requires further functional validation to elucidate their biological significance.

From a broader perspective, this study highlights the value of multi-omics approaches in uncovering complex biological responses that would remain hidden in single-layer analyses. The integration of transcriptomics, metabolomics, and lipidomics enables the identification of coherent biological patterns and provides a more comprehensive understanding of the mode of action of bioactive compounds.

Finally, these findings reinforce the potential of agro-industrial by-products such as red onion peels as sources of functional ingredients with systemic biological effects. Beyond their antioxidant properties, these extracts appear to modulate fundamental pathways involved in cellular adaptation, positioning them as promising candidates for applications in health and nutrition.

Although the present study was not designed to establish causal molecular mechanisms, the integration of transcriptomic, metabolomic, and lipidomic data suggests that the observed effects are driven by coordinated biological adaptations associated with oxidative stress resistance. The convergence of changes across multiple omics layers supports the hypothesis that polyphenol-rich red onion peel extracts promote cellular homeostasis through the modulation of stress-response pathways, metabolic adaptation, and membrane lipid composition.

Despite the comprehensive multi-omics characterization presented in this study, some limitations should be acknowledged. Although the integrated transcriptomic, metabolomic, and lipidomic analyses revealed coordinated molecular responses associated with treatment, the present study was not designed to establish causal relationships between the identified pathways and the observed biological effects. Therefore, additional functional studies will be required to validate the role of the proposed molecular mechanisms and to confirm the biological relevance of the identified biomarkers. Future research should focus on targeted validation of key genes, metabolites, and lipid species, as well as on evaluating the translational potential of these findings in additional experimental models.

## 5. Conclusions

Polyphenols are naturally occurring compounds with well-established antioxidant properties, and red onion (*Allium cepa*) peels represent a rich and underutilized source of these bioactive molecules. In this study, we demonstrated that polyphenol-rich extracts derived from red onion peels induce coordinated molecular responses in *C. elegans* under oxidative stress conditions, as revealed by an integrative multi-omics approach.

The combined transcriptomic, metabolomic, and lipidomic analyses revealed molecular adaptations associated with stress response, metabolic reprogramming, and lipid remodeling, supporting a model of cellular adaptation to oxidative stress. Importantly, the observed effects suggest that the extract’s biological activity extends beyond direct antioxidant action and involves the modulation of endogenous adaptive mechanisms.

Although no differences in survival were observed under the tested conditions, the multi-omics signatures indicate the activation of molecular pathways associated with oxidative stress adaptation. While the biological interpretation was strengthened by the concordance observed across transcriptomic, metabolomic, and lipidomic datasets, independent validation of selected differentially expressed genes by RT-qPCR would further reinforce the robustness of the transcriptomic findings and should be considered in future studies.

Overall, our findings highlight the potential of agro-industrial by-products as sustainable sources of functional ingredients and demonstrate the value of multi-omics approaches for elucidating the mode of action of complex bioactive extracts.

## Figures and Tables

**Table 1:** Total phenolic content and antioxidant power of each extract, measured by the Folin–Ciocalteu and DPPH assays, respectively. The results are presented as mean ± standard deviation (SD). Each value represents the mean of two independent analytical measurements.

Sample	TPC ± SD (mg GAE/g)	DPPH ± SD (mg GAE/g)
PLX 340	404.37 ± 13.10	190.58 ± 4.74

**Table 2:** Polyphenolic composition of the enriched extract. The results are presented as mean ± standard deviation (SD). Each value represents the mean of two independent analytical measurements.

PLX 340—Red Onion Peels Enriched Extract	
Metabolite	Concentration (mg/g)
Protocatechuic acid	22.56 ± 0.87
Quercetin	122.32 ± 2.57
Isorhamnetin	0.58 ± 0.11
Tamarixetin	0.44 ± 0.07
Quercetine-7-O-glucoside	2.28 ± 1.14
Isoquercitrin	1.75 ± 0.79
Spiraeoside	61.10 ± 1.14

## Data Availability

The original contributions presented in this study are included in the article/Supplementary Materials. Further inquiries can be directed to the corresponding authors.

## Supplementary Materials

The following supporting information can be downloaded at https://www.mdpi.com/article/10.3390/biom16071024/s1, Table S1: Time-dependent accumulation of intracellular ROS in *C. elegans* exposed to 35 °C; Table S2: Raw and normalized transcriptomic counts for test and control conditions in *C. elegans* (Excel file); Table S3: ^1^H NMR-based metabolite concentrations in control and test samples (Excel file); Table S4: Comprehensive lipidomics dataset for test and control samples (Excel file).

## Abbreviations

ART	Adsorption resin techniques
*C. elegans*	*Caenorhabditis elegans*
DG	Diacylglycerols
DPPH	2,2-diphenyl-1-picrylhydrazyl
LC-MS/MS	Liquid Chromatography-Tandem Mass Spectrometry
MA	Minus-Average plot
MS/MS	Tandem Mass Spectrometry
NGM	Nematode Growth Medium
NGS	Next-Generation Sequencing
NMR	Nuclear Magnetic Resonance
PC	Phosphatidylcholines
PCA	Principal Component Analysis
PE	Phosphatidylethanolamines
RNA-Seq	RNA sequencing
SM	Sphingomyelins
sPLS	Sparse Partial Least Squares
SWE	Subcritical Water Extraction
TG	Triacylglycerols
TPC	Total Phenolic Content
UPLC-MS/MS	Ultra-Performance Liquid Chromatography-Tandem Mass Spectrometry
UHPLC-qTOF	Ultra-High-Performance Liquid Chromatography coupled to Time-of-Flight Mass Spectrometry
